# Sclerodermic Cardiomyopathy—A State-of-the-Art Review

**DOI:** 10.3390/diagnostics12030669

**Published:** 2022-03-09

**Authors:** Adrian Giucă, Tea Gegenava, Carmen Marina Mihai, Ciprian Jurcuţ, Adrian Săftoiu, Diana Monica Gȋrniţă, Bogdan Alexandru Popescu, Nina Ajmone Marsan, Ruxandra Jurcuț

**Affiliations:** 1 Department of Cardiology, “Prof. Dr. C.C. Iliescu” Emergency Institute for Cardiovascular Diseases, 022328 Bucharest, Romania; adriangiuca17@gmail.com (A.G.); bogdan.a.popescu@gmail.com (B.A.P.); 2 Department of Research Methodology, Craiova University of Medicine and Pharmacy, 200349 Craiova, Romania; adriansaftoiu@gmail.com; 3 Department of Cardiology, Leiden University Medical Center, 2300 RC Leiden, The Netherlands; t.gegenava@lumc.nl (T.G.); n.ajmone@lumc.nl (N.A.M.); 4 Department of Internal Medicine, Tbilisi State Medical University, Tbilisi 0159, Georgia; 5 Department of Rheumatology, University Hospital Zurich, University of Zurich, 8091 Zurich, Switzerland; carmenmarinamihai@yahoo.com; 6 Department of Internal Medicine, “Carol Davila” Central Military Emergency University Hospital, Calea Plevnei no 134, 010825 Bucharest, Romania; cjurcut@gmail.com; 7 Division of Immunology, Allergy & Rheumatology, College of Medicine, University of Cincinnati, 231 Albert Sabin Way, ML 0563, Medical Science Bldg. (MSB), Rm 7409, Cincinnati, OH 45267-0563, USA; girnitdm@ucmail.uc.edu; 8 Faculty of Medicine, “Carol Davila” University of Medicine and Pharmacy, 020021 Bucharest, Romania

**Keywords:** systemic sclerosis, cardiomyopathy, echocardiography, speckle-tracking, strain, cardiac magnetic resonance

## Abstract

Systemic sclerosis (SSc) is a chronic autoimmune disorder with unknown triggering factors, and complex pathophysiologic links which lead to fibrosis of skin and internal organs, including the heart, lungs, and gut. However, more than 100 years after the first description of cardiac disease in SSc, sclerodermic cardiomyopathy (SScCmp) is an underrecognized, occult disease with important adverse long-term prognosis. Laboratory tests, electrocardiography (ECG) and cardiovascular multimodality imaging techniques (transthoracic 2D and 3D echocardiography, cardiac magnetic resonance (CMR), and novel imaging techniques, including myocardial deformation analysis) provide new insights into the cardiac abnormalities in patients with SSc. This state-of-the-art review aims to stratify all the cardiac investigations needed to diagnose and follow-up the SScCmp, and discusses the epidemiology, risk factors and pathophysiology of this important cause of morbidity of the SSc patient.

## 1. Introduction

Systemic sclerosis (SSc) is a rare autoimmune disorder characterized by the following triad: microvascular damage, initial innate and adaptive immune response alterations, and further development of fibrosis in skin and several internal organs [1]. SSc is more prevalent in females than in males with a ratio of 3–14:1, with symptom onset age peaking between 30 and 55 years [1,2,3].

The cutaneous involvement in SSc can be either limited (lcSSc) or diffuse (dcSSc), with major differences regarding skin changes, the extent and rate of disease progression, visceral lesions, immunologic profile, and prognosis. LcSSc patients are characterized by skin thickening limited to the face, neck, and extremities distal to knees and elbows [4]. On the other hand, in dcSSc, skin fibrosis involves at least one proximal region (thorax, abdomen, back, and proximal segments of the limbs) [4]. While in lcSSc, visceral damage is mostly limited and quite stable over time, with Raynaud’s phenomenon (RP) preceding the development of the systemic disorder for years, dcSSc associates in most cases rapid and severe skin and internal organ involvement [4].

Even though certain systems are actively screened for the presence of the disease, including pulmonary fibrosis and pulmonary hypertension, SSc-related myocardial damage is an underrecognized entity with an incomplete understanding of the pathogenic mechanisms, long-term sequelae, and prognosis [5]. What we will further call sclerodermic cardiomyopathy (SScCmp) is characterized by the presence of contractile dysfunction, either systolic or diastolic, frequently subclinical, myocardial fibrosis as well as conduction and rhythm disturbances, all generating high rates of adverse clinical outcomes. This review aims to define the multiple aspects of SScCmp and also present the different cardiovascular imaging modalities which can be employed for its diagnosis.

## 2. Pathophysiology

The complexity of SSc is reflected by its pathogeny. The current concept is that external factors, acting on a genetically susceptible host, trigger a chronic, multifocal, auto amplifying cascade with early vascular damage, inflammation, and autoimmunity, followed in more advanced stages by fibrosis—the trademark of SSc. Accumulation of collagen, elastin, glycosaminoglycans, and fibronectin leads to permanent tissular scarring generating compact and rigid connective tissue [1]. Abnormal vascular reactivity is secondary to the imbalance between vasodilator and vasoconstrictor factors generated from the injured endothelium and defective angiogenesis [2].

Historically, in 1926 Heine performed the first description of cardiac disease in SSc using autopsy and describing abnormalities in the coronary arteries, myocardium, and pericardium [6]. He was followed by Weiss and Warren, who examined nine patients with SSc and congestive heart failure (CHF) and warned on the presence of a distinct SSc-related cardiac disease, which should not be attributed to atherosclerosis, hypertension (HTN), rheumatism, syphilis, or congenital heart disease [7,8,9].

Primary cardiac involvement directly involves the myocardium, pericardium, valvular structures, coronary arteries, and conduction system, and is determined by myocardial fibrosis, autonomic neuropathy, and small vessel disease [10,11,12,13]. Direct valvular damage is the rarest type of manifestation [14].

Primary ultrastructural alterations in the myocardium are most probably multifactorial: micro- and/or macrovascular changes are supposed to lead to irreversible damage through fibrosis [10]. In 1976, Bulkley et al. made the first description of certain focal lesions, from contraction band necrosis to fibrosis, with disposition not to coronary artery disease (CAD), suggesting that this may rather be caused by ischemic necrosis and intermittent reperfusion after vascular spasm [15,16].

Cardiac RP is another possible explanation for the myocardial damage in SSc, as Alexander et al. identified cold, physical stress, and pharmacologic stress-induced myocardial perfusion defects by means of positron emission tomography (PET-CT), single-photon emission computed tomography (SPECT) and cardiac magnetic resonance (CMR) [15,17]. Using contrast echocardiography, Mizuno et al. evaluated 51 patients with SSc with normal myocardial flow at rest but with cardiac RP after cold-provocation. They showed that it is a very important predictor for future left ventricle (LV) contractile dysfunction [18,19]. The absence of severe RP identified cases that did not exhibit systolic dysfunction and adverse myocardial remodeling [20].

Long-lasting vascular disease leads to generation of the other important, virtually irreversible aspect of the SSc-related cardiac disease: myocardial fibrosis, which is disposed in the interstitium, does not involve the immediate subendo/epicardial layer and is equally distributed among both ventricles, being linked with contractile band necrosis following repeated ischemia-reperfusion events [12,13]. The fundamental difference between ischemic and SSc fibrosis is that the latter does not associate myocardial hemosiderin deposits [21].

The multitude of potential tissular targets of the systemic disease may lead to polymorphic clinical expression: myocarditis, myocardial fibrosis, restrictive cardiomyopathy (RCM), systolic/diastolic ventricular dysfunction, heart failure (HF), valvular regurgitation, CAD, rhythm and conduction disturbances, pericardial disease (effusion, fibrosis) may all be seen [10,11,22,23,24].

## 3. Traditional Cardiovascular Risk Factors in SSc

In patients with SSc, conventional cardiovascular risk factors (CVRF) and comorbidities like HTN, CAD, and dyslipidemia appear to have similar frequencies as in the general population [21]. In an angiographic study conducted on 172 patients with SSc with suspected CAD, Steen et al. found that the prevalence of significant atherosclerotic coronary disease is similar when compared to symptoms-, age-, and sex-matched controls [3,25]. Recent studies suggest there is an SSc-related increase in cardiovascular risk, so patients with SSc need to be actively screened and corrected for traditional CVRF to limit the potential subsequent myocardial injury [26,27].

## 4. Risk Factors for Primary Myocardial Disease in SSc

Data obtained by multiple regression analysis from the European Scleroderma Trials and Research Group (EUSTAR) Database have shown that the following are considered to be risk factors for primary myocardial involvement: advanced age, rapid progression of skin thickening, male gender, dcSSc, duration of disease, digital ulcers, simultaneous renal and muscular impairment, disease activity score, pulmonary fibrosis, pulmonary arterial hypertension (PAH) [1,24,28]. Meune et al. compared 129 SSc patients with left ventricle ejection fraction (LVEF) < 55% with 256 SSc patients and LVEF ≥ 55% and found the following independent factors associated with LV dysfunction: male sex (OR: 3.48; 95% C.I. 1.74–6.98), age (OR: 1.03; 95% C.I. 1.01–1.06), digital ulcers (OR: 1.91; 95% C.I. 1.05–3.5), and myositis (OR: 2.88; 95% C.I. 1.15–7.19), while calcium channel blockers (CCB) appeared to have a protective effect (OR: 0.41; 95% C.I. 0.22–0.74) [24].

Using scintigraphy, Steen et al. showed that physical stress-induced perfusion defects predicted the occurrence of cardiac disease or death [29]. Cardiac RP is an independent factor associated with the development of LV systolic dysfunction and ventricular adverse remodeling [20].

Disease-specific autoantibodies offer important prognostic information in patients with SSc. The presence of anti-topoisomerase I (anti-Scl70), anti-Ku, anti-U3 RNP, anti-histone, and anti-ribonucleic acid (RNA) polymerase (I, II, III) antibodies are associated with a higher risk of SSc-related cardiac disease [30]. In addition, serum levels of interleukin 6 (IL-6), tissue inhibitor matrix metalloproteinase (TIMP) 1, and TIMP 2 correlate with the degree of LV diastolic dysfunction [23,30].

Associated with more severe internal organ damage and an immunologic profile dominated by the presence of anti-Scl70 antibodies, dcSSc causes a higher rate of severe complications [31]. However, myocardial injury might be found in lcSSc as well [31]. In a large epidemiologic Italian study, symptoms attributed to heart disease were more often seen in dcSSc (32%) than in lcSSc (23%), but the difference did not reach statistical significance [32]. In the registry of the German Network for Systemic Scleroderma, overall higher rates of cardiac involvement were seen in dcSSc [33]. Concordantly, Ostojić et al. demonstrated that heart involvement characterized 29.1% of the dcSSc cases analyzed by them, compared to only 8% seen in lcSSc (*p* < 0.001) [34]. An echo study revealed LV anomalies in 18/57 patients with lcSSc, in comparison to only 5/23 with dcSSc [35].

Finally, more severe nailfold capillaroscopy findings have been demonstrated to associate with heart involvement in patients with SSc [36]. A reduced capillary density was found to be associated with lower functional status (evaluated by New York Heart Association (NYHA) class) and with electrocardiographic (ECG) abnormalities (present in 25–75% SSc cases) [37,38,39]. Moreover, Caramaschi et al. demonstrated an increase in cardiac disease frequency as capillaroscopic patterns progress [38].

## 5. Myocardial Involvement in SSc

Myopericardial inflammation, perfusion defects, and fibrosis are the major underlying factors in the SScCmp [40]. Myocardial inflammation has been documented histologically in patients with SSc and recent onset of cardiac disease and it is considered a risk factor for myocardial dysfunction [40]. Irrespective of skin involvement, myocardial injury appears to be frequent, early and rapidly progressive, as shown by Perera et al., who found that anti-Scl70 antibodies, alongside intermediate or rapid progression of cutaneous thickening, are risk factors for early cardiac involvement: 41% of patients classified as having rapid progression had cardiac impairment, of which 75% developed it in the first 3 years from the onset of the skin disease [24,31,41].

By using CMR, Rodriguez-Reyna et al. identified myocardial fibrosis in 45% of 62 investigated SSc patients, and it was more prevalent in the dcSSc subtype [42]. After analyzing 36 SSc cases, Muresan et al. found that 60% had conduction disturbances, with no difference between the disease subtypes [43].

Despite having a high frequency of primary myocardial disease, SSc patients most frequently present with occult, asymptomatic initial disease [11,12,15,21,44]. While only approximately 10% of all patients with SSc have symptoms suggesting myocardial involvement, necroptic studies identified heart muscle disease in 12–89% of SSc patients [12,45].

As mentioned earlier, being a systemic disease, SSc can generate signs and symptoms that can be falsely attributed to extracardiac pathology [11,21]. The most frequent complaints are represented by exertional dyspnea and fatigue [21]. Chest discomfort, when present, tends to be generated by pericarditis, but it can also be the consequence of myocardial ischemia, occurring either because of microvascular disease, or because of atherosclerosis of epicardial coronary arteries [21]. Irregular heartbeats, dizziness, syncope, or sudden cardiac death (SCD) have also been reported [21].

## 6. Cardiac Investigations in SSc-Related Myocardial Involvement

The diagnosis of SSc-related myocardial involvement is not always straightforward, as it is intricated with other possible etiologies if concurrent CVRF are present. However, a multimodal diagnostic approach of myocardial alterations, not explained solely by CAD or significant valvular heart disease, will lead to a diagnosis of SScCmp.

A summary of all cardiac investigations useful in assessing the cardiac injury in SSc is listed in Table 1.

### 6.1. Cardiac Biomarkers

An ideal biomarker should be defined by high sensitivity, specificity, and reproducibility. The N-terminal pro B-type natriuretic peptide (NTproBNP) fulfills these criteria, as it is cheap, reliable, and easy to detect through non-invasive procedures [23]. Its serum level can be used as a first-line tool to stratify for risk of myocardial damage and heart failure and to follow-up patients [23]. Allanore et al. demonstrated the additive value of NTproBNP alongside echocardiography and Tissue Doppler Imaging (TDI) for the identification of depressed myocardial contractile function [22]. Schioppo et al. found increased serum levels of NTproBNP in 27/87 (31%) patients with SSc with known cardiac dysfunction, with values significantly higher when compared to patients without cardiac dysfunction (*p* = 0.0003; 95% C.I.: 57–232) [23]. Using a cut-off value of 130 pg/mL, a sensitivity of 74%, specificity of 70%, negative predictive value (NPV) of 85% were obtained for detecting impairment of cardiac function [23]. On the other hand, it appears that for detecting heart abnormalities in the SSc population, lower cut-off values should be used for B-type natriuretic peptide (BNP) and NTproBNP [28]. Serum NTproBNP did not vary across different skin subtypes [23].

Cardiac troponins (cTn) are sarcomeric regulatory proteins with high specificity for myocardial tissue. According to Barsotti et al., high levels of high-sensitivity cTn (hs-cTn) are positively correlated with cardiac disease in SSc, but with low specificity and sensitivity (83%, 66.7%) [46].

### 6.2. ECG Changes

Myocardial tissue anisotropy is defined by the increased spatial dispersion of ventricular repolarization and plays a role in arrhythmogenesis [30]. The severe fibrotic process characteristic of SSc leads to direct damage of the cardiomyocytes. These changes will reflect in the cell’s fundamental ability to generate electrical impulses so that subtle anomalies which indicate myocardial injury may be detected by ECG. The deposit of collagen fibers between cardiac cells forms fibrosis islands, which in return generate electromechanical abnormalities by interrupting normal intercellular connections, this being a substrate for reentry circuits and ectopic automaticity [30]. In addition, the obliterative vasculopathy will lower the oxygen supply towards the myocardium, further aggravating the events [30].

An abnormal ECG tracing (supraventricular or ventricular arrhythmias, conduction disturbances, ST-T changes) is present in 25–75% of patients with SSc and it independently predicts mortality [39]. In addition, an anteroseptal myocardial infarction pattern can be seen on resting ECG in 10% of patients [30].

The most relevant ECG change is represented by arrhythmias, as according to data from the 2010 European League Against Rheumatism (EULAR)/EUSTAR, 5–6% of total deaths in SSc population are attributable to arrhythmia [30,59]. In the Genetics Versus Environment in Scleroderma Outcome (Genisos) Study, the presence of ventricular arrhythmia carried a 2.18 RR of SCD compared to patients free from it [60]. According to Bienias et al., 36% of SSc cases exhibit premature ventricular beats (PVB) (couplets) and non-sustained ventricular tachycardia (NSVT) [61]. The highest probability of abnormal electric events is within the first 3 years of progression of the systemic disease, but even after 6 years, their frequency remains high [30]. Moreover, the high frequency of PVB (up to 67% of SSc patients in a cohort) correlated with mortality and SCD risk [48]. However, SCD is rather rare, as from a total number of 391 deaths in 1258 patients with SSc, only 18 (5%) suffered this outcome [62].

Even though the highest clinical impact is generated by ventricular arrhythmias, data obtained by Ferri et al. through 24-h Holter ECG monitoring demonstrate that supraventricular arrhythmias are also frequent, detected in 66% of SSc patients [47]. Transient atrial fibrillation, flutter, or reentrant supraventricular tachycardia are described in 20–30% of cases [63]. Furthermore, cardiac decompensation in SSc is frequently associated with arrhythmia. De Luca et al. found frequent PVB in 24/100 patients with recently diagnosed HF, PVB correlating positively with hs-cTn and negatively with LV systolic function [49].

The presence and/or severity of arrhythmia does not appear to correlate with the skin subtype or the occurrence of signs/symptoms [39]. Muresan et al. identified by CMR significant myocardial fibrosis in more than 80% of SSc patients, and more than 60% exhibited significant ventricular arrhythmia; however, there was no correlation between the arrhythmic burden and myocardial fibrosis, and diffuse fibrosis was the only pattern that influenced the number of PVB [43]. In a larger prospective study, CMR-derived T2 ratio (as a marker of edema) and the extent of late gadolinium enhancement (LGE) (as a marker of myocardial scar) had the greatest utility as independent predictors of rhythm disturbances in SSc patients [64].

De Luca et al. demonstrated that ventricular arrhythmias aggravate the SSc patient’s prognosis, as more than 1190 PVB/24 h associate a sensitivity of 100% and specificity of 83% in predicting SCD or later necessity of implantable cardioverter defibrillator (ICD) implantation [49].

To identify sensitive ECG markers for arrhythmic risk stratification, Ciftci et al. examined QT dynamicity (QTd) and heart rate variability in SSc patients [65]. QTd is the slope of the linear regression line of QT/RR value, and it is a known predictor of ventricular arrhythmia in patients with QT prolongation [65]. The Arrhythmogeneity Index (ratio of Tpe–T-wave peak-to-end-interval and QTc interval) is a global predictor of SCD and ventricular arrhythmia, Okutuku et al. finding a correlation between it and the modified Rodnan Skin Score (mRSS) in 107 SSc cases [50].

### 6.3. Echocardiographic Assessment of Primary Myocardial Disease in SSc

In the comprehensive process of imaging the SScCmp, echocardiography plays a key role. Reproducibility, large-scale availability, and high sensitivity are key attributes that propose this imaging method as a standard investigation for characterizing and following-up SSc heart disease [14].

It is recommended that the patient with SSc should be actively screened by means of two-dimensional transthoracic echocardiography (2DTTE) annually in order to assess systolic and diastolic LV function and systolic pulmonary arterial pressure (sPAP) [66].

#### 6.3.1. LV Systolic Function

In the SSc population, LV systolic dysfunction is well described from an epidemiologic viewpoint [53]. Data from EUSTAR, which are derived from the analysis of 7073 cases (mean age of 56 ± 14 years), demonstrated a prevalence of 5.4% for reduced LVEF (<50/55%, depending on the laboratory) [52]. On the other hand, a multicentric French study on 570 SSc patients reported a 1.4% prevalence for LV systolic dysfunction, significantly lower than EUSTAR [67]. However, it appears that in exercise, the frequency of contractile LV dysfunction is high (46%) [4].

While the classical RP affects the extremities of almost all SSc patients, it is postulated by some investigators that a similar cold- or stress-induced excessive vasoconstriction may be an important cause of diffuse damage in internal organs [17,68,69]. Alexander et al. studied 13 patients with SSc (11 female patients, mean age 38 years, mean disease duration of 6 years), assessing the dynamic influence of cold exposure of the extremities on myocardial contractility, using exposure to cold for 2 min and comparing them with healthy controls [17]. In total, 12/13 patients manifested spontaneous LV reversible hypokinesis, starting 30–40 s after exposure and completely remitted after an interval of 2–4 min; the contractile dysfunction was localized especially in the anterolateral and inferoposterior regions of the LV [17]. In order to confirm the hypothesis of a visceral RP, the authors also quantified the myocardial perfusion using thallium scintigraphy, and 10/13 cases had transitory defects in radionuclide uptake and at least one LV kinetic abnormality during cold exposure [17]. The association between cold-induced myocardial perfusion defects and functional anomalies was significant, as all patients with regional impairment in radionuclide uptake had an anatomical correlation with the area of LV segmentary dysfunction [17]. Maybe the most illustrative case is the one of a 33 year-old Caucasian female with unilateral cervical sympathectomy for severe RP [17]. When the hand contralateral to the surgical intervention was exposed to cold, perfusion defects could be observed at septal and inferoapical level, with concordant contractile dysfunction detectable by echocardiography, initiated 20 s after stimulus application [17]. Repeating the procedure on the upper limb ipsilateral to the sympathectomy, it generated only a slight transitory defect in myocardial perfusion [17]. Using contrast-enhanced echocardiography, Mizuno et al. published more data regarding cardiac RP by investigating 51 patients with SSc who had normal myocardial flow at rest and developed segmentary dysfunction after cold provocation test, which is a strong predictor for future global alteration of myocardial function [19]. The anatomical correlation between transitory defects in thallium uptake and wall motion abnormalities suggests that regional myocardial ischemia is related to ventricular dysfunction by spasm of small coronary arteries [17]. However, evaluating the systolic function of the LV in the SSc population only by means of measurement of LVEF is not sensitive enough as it detects cardiac dysfunction in only 5% of cases [52].

The myocardium is composed of fibers with longitudinal, circumferential, and radial disposition and the subendocardial layer is the structure with maximum susceptibility to ischemia. Its fibers are mostly arranged in a longitudinal manner, so longitudinal contractile dysfunction is one of the earliest signs of myocardial damage [28]. Therefore, studying longitudinal myocardial deformation using strain imaging is a more sensitive technique than standard echocardiographic for describing cardiac deformation, particularly useful in the study of fibrous sequelae in SSc [14,24]. Used in conjunction with 2DTTE, strain imaging by speckle-tracking echocardiography (STE) offers a precise measurement of regional systolic function, being an operator and angle independent [14].

Assessing 113 SSc cases (51 lcSSc, 53 dcSSc), Yiu et al. demonstrated that in this population, subtle LV contraction abnormalities are present before routinely used global echocardiographic parameters become abnormal (i.e., LV cavitary dimensions, LVEF) [70]. In their study, LV global longitudinal strain (GLS) and circumferential strain rates have been independently associated with the patient’s functional capacity (assessed by cardiopulmonary exercise test (CPET)) and the presence of ventricular arrhythmias (documented by 24-h ECG Holter monitoring) [70]. Regarding SSc skin subtypes, the authors discovered that in dcSSc, as well as in lcSSc, GLS and global circumferential strains were modestly, but significantly, reduced compared to controls, with more important reductions in the former than the latter [70].

Van Wijngaarden et al. were the first authors to demonstrate many of the 234 SSc cases (165 lcSSc, 69 dcSSc, 196 female gender) that serial measurements with STE can detect progressive impairment of LV systolic function in a relative short time frame [71]. During the follow-up period, 19% of patients exhibited a reduction of ≥15% in GLS, associated with proximal muscle weakness, lung fibrosis, renal comorbidity, the elevated value of NTproBNP, and all-cause mortality [71].

Another recent study from this group proved that from all echocardiographic parameters used, LV GLS was the only one independently associated with the combined endpoint (all-cause mortality and hospitalizations for heart failure, myocardial infarction, coronary interventions, device implantations, arrhythmias, cerebral infarction, and peripheral ischemic disease) in an SSc population with a similar mortality rate (9%) and follow-up as the EUSTAR database [72]. Moreover, a combined patient approach including determination of NTproBNP, alongside LV GLS is useful in risk stratification, but more importantly, it might be able to exclude the presence of SScCmp [72].

In order to better understand the early contraction abnormalities generated by the fibrotic process, D’Andrea et al. analyzed the association between LV function, coronary flow reserve (CFR) and endothelial function in 33 asymptomatic SSc patients (18 dcSSc and 15 lcSSc), compared with 30 healthy controls [12]. Standard echo measurements did not find any disparities between LVEF and LV diameters in the two groups, but regional systolic function evaluated by strain rate showed reduced values at interventricular septum level and LV lateral wall, indicating a direct and early damage, at a time when other global and regional indicators did not show any abnormalities [12]. Moreover, CFR and brachial flow-mediated vasodilatory response were directly and independently related to the extension and severity of the myocardial dysfunction [12]. As a consequence, the impairment of endothelial function in the SSc patient, probably explained by nitrate tolerance because of augmented nitric oxide (NO) basal production, may contribute to LV systolic and diastolic dysfunction [12].

The evaluation of myocardial electromechanical proprieties is a new approach for detecting latent heart disease and it is based on the existence of a heterogenous prolongation of the time from electrical impulse generation to mechanical contraction of the ventricle [3]. In SSc, LV segments are diffusely deteriorated, with different degrees of severity, secondary to extended fibrosis and segmentary systolic dysfunction alongside contraction dyssynchrony, are considered results of this association [3].

#### 6.3.2. LV Diastolic Function

In contrast to systolic dysfunction, the incidence, risk factors, and effects of altered diastolic function in SSc patients are less studied [53].

Even though heart failure with reduced ejection fraction (HFrEF) is a well-established clinical entity with adverse clinical outcomes, heart failure with preserved ejection fraction is less studied (HFpEF) but determines mortality and morbidity rates similar to the ones generated by HFrEF [73]. Diastolic dysfunction (DD) represents the hallmark of HFpEF and is a fundamental part of the assessment of the SSc patient. Myocardial fibrosis impairs the relaxation of the ventricles, which can be used as an early indicator of the disease [53]. DD is more prevalent in SSc than in healthy subjects, and it was present in 101/570 cases (17.7%) [22,67]. Other studies report frequencies ranging from 17 to 62%, which is 4–5 times more frequent than systolic dysfunction [22,53,67,74,75].

Tennoe et al. showed in a large and unselected SSc cohort (275 SSc cases) that baseline frequency of DD was 17% (15% grade I DD, 54% grade II DD, and 5% grade III DD), and after a median follow-up of 3.4 years, it increased to 29% [76]. Risk factors for baseline DD were: age, NTproBNP, pulmonary hypertension, and calcinosis [76]. Furthermore, the authors documented higher mortality rates in patients with SSc and DD (26 cases of 46; 57%) compared to subjects with normal diastolic function, and higher death rates (8 cases of 26; 31%) for patients with newly diagnosed DD during the follow-up period [76].

Hinchcliff et al. studied 153 patients with SSc (85% females), between 2005 and 2009, with a mean age of 51 ± 13 years, 60% lcSSc, and detected systolic dysfunction in 8/153 and diastolic dysfunction in 35/153 [53]. S’ tissue Doppler velocities have been independently associated with LV diastolic dysfunction and e’ Doppler velocities, and as the disease duration increased, longitudinal dysfunction occurred, both in systole and diastole, with progressive deterioration [53]. After multivariate analysis, risk factors for low e’ velocities were: duration of systemic disease, age, CAD, HTN [53]. Neither cutaneous subset, nor disease severity (quantified by mRSS) were related to diastolic impairment [53]. Moreover, the baseline value of e’ velocity predicted mortality risk and every standard deviation in its decrease elevated the mortality rate by 3.2 times, so measuring e’ can be useful in identifying subclinical myocardial dysfunction [53].

Candell-Riera et al. published results of a comprehensive analysis based on non-invasive imaging techniques, conducted on 63 patients with lcSSc cases (51 females) and mean age of 53.8 ± 11.8 years [45]. The most important echocardiographic abnormalities were: diastolic dysfunction in both ventricles, mild mitral regurgitation, and thickening of subvalvular mitral apparatus [45]. The change in LV diastolic parameters did not correlate with the patient’s age, blood pressure, heart rate, mitral regurgitation, or pericardial disease [45,77].

LV myocardial early diastolic peak velocity presented low values at the basal segments of the interventricular septum, but also in the middle and basal segment of the LV lateral wall [12]. Measuring tissular velocities at the plane of mitral and tricuspid annulus generates a higher rate of detecting myocardial damage, and because tissue Doppler evaluation is widely available, it should be part of the standard echocardiographic evaluation in SSc [24].

#### 6.3.3. Ventricular-Arterial Coupling (VAC)

Even though the end-diastolic LV diameter, fractional shortening and LVEF are routinely used parameters for the 2D echocardiographic evaluation of SSc patients, they are load-dependent and do not systematically reflect the myocardial contractile status [51]. VAC is a novel measurement that is based on the complex interplay between the cardiac function and arterial system and is a major determinant of global cardiovascular performance [78]. It is defined by the ratio between arterial elastance (Ea) and end-systolic LV elastance (Ees) [51]. Therefore, VAC uncoupling may be able to predict morbi-mortality in different pathogenic entities, including SSc, because regulating processes from the extracellular matrix and cytoskeleton are complex biochemical pathways that simultaneously damage both the structure and function of the heart and arteries by means of fibrosis deposits [51,78]. Tona et al. are the first to have conducted a study that evaluated the relationship between LV pressure-volume status and VAC by three-dimensional TTE (3DTTE) in SSc [51]. The advantages of 3DTTE reside in the superior accuracy, precision, and reproducibility for volume measurements, and as such, for measurement of VAC [51]. The study included 65 SSc patients and corresponding matched controls. Published results demonstrated that 3D derived VAC may be significantly higher in dcSSc compared to lcSSc, despite normal LVEF and worsen as disease severity increased [51]. Moreover, it seems that VAC may be able to predict the risk of major adverse cardiovascular events (MACE) in the SSc population [51]. Traditional parameters used for routine assessment of the LV (LV end-diastolic volume (LVEDV), LV end-systolic volume (LVESV), LVEF) had similar values between patients and controls, with the mention that the stroke volume (SV) of the LV tended to be slightly lower than in healthy subjects (*p* = 0.07) [51]. Values of Ea and Ees were significantly higher in the SSc population, thus VAC was similar between the two groups. Moreover, diastolic elastance (Eed) had higher values in the patient group, indicating high filling pressures, which represent the hallmark of one of the most frequent conditions associated with SScCmp-HFpEF [51].

Also, the authors document a possible intrinsic LV systolic dysfunction which is caused by the inability of the LV to compensate for the elevated afterload, which may be caused by the increased arterial stiffness because of collagen deposits; this, in turn, leads to high values of Ea and is associated with adverse outcomes [51]. They conclude by highlighting the fact that the myocardium of the SSc patient is unable to cope with the high afterload, which in turn leads to long-term adverse outcomes [51].

#### 6.3.4. Right Ventricle (RV) Evaluation

With a thin free wall, three-dimensional complex geometry, and a continuous link with the pulmonary system, the RV poses certain challenges for the accurate evaluation of its internal chamber dimensions and changes during the cardiac cycle [54]. In the setting of systemic diseases, it is often involved, either by direct extension of the lesional process or by afterload changes and ventricular interdependence generated by anatomic proximity with the LV [54].

In SSc, for direct echocardiographic evaluation of RV myocardial function, several parameters can be assessed: tricuspid annular plane systolic excursion (TAPSE), free wall tissue Doppler S’, RV fractional area change (RVFAC), RV ejection fraction (RVEF) using 3DTTE, sPAP, and mean pulmonary arterial pressure (mPAP) estimates. More recently a non-invasive marker of pulmonary vascular resistance—the ratio of tricuspid regurgitant velocity to pulmonary artery velocity–time integral—has been proposed [28].

SSc determines early RV dysfunction, strongly associated with the degree of skin and pulmonary involvement, but also with the serology (anti-Scl70 antibodies correlate with early RV DD) [12,54].

In order to better characterize the primary myocardial damage of the RV, Kahan et al. conducted a study on 42 patients with SSc with normal sPAP and disease duration of less than 5 years, observing that RVEF correlated with LVEF and peak filling rate, while the alteration of pulmonary function or sPAP did not influence RV’s function, strongly implying the possibility of an intrinsic myocardial disease [31].

Primary myocardial disease is directly caused by two factors: the fibrotic process and the coronary microvascular disease, with global diastolic function impairment alongside reduced CFR seen even in asymptomatic cases [54]. Experimental studies demonstrate that the RV, unlike the LV, starts the ejection phase after a minimal isovolumetric contraction, and the diastolic filling is performed in the absence of isovolumetric relaxation, since it pumps against a lower vascular resistance [54]. To characterize the systolic and diastolic function of the myocardium and their relationship with features of the systemic disease, D’Andrea et al. compared 23 asymptomatic SSc patients (11 dcSSc, 12 lcSSc) (using Doppler myocardial imaging (DMI), strain rate (SR), and strain) with 25 similar age and sex controls [54]. Significantly lower early diastolic peak velocities were observed at the plane of the tricuspid annulus, alongside a prolonged relaxation time, despite only slightly reduced Doppler measurements, suggesting that SSc patients suffer from early RV diastolic dysfunction [54]. Regional systolic function evaluated by pulsed DMI presented normal systolic peak velocities, with prolongation of pre-ejection times at the tricuspid annulus, even after age and heart rate correction [54].

Despite normal LVEF, TAPSE, and DMI systolic peak velocities, strain and SR were significantly low at the site of both free walls [54]. Supplementary, RV peak Em velocity was the only independent predictor of mRSS, sPAP, and pulmonary fibrosis, with values lower than 0.11 m/sec selecting cases that suffered from worse cutaneous and pulmonary disease, with high sensitivity and specificity [54].

Even though, in the recent years, many 2D and Doppler echocardiographic parameters have been proposed for the evaluation of RV contractile function, most of them are limited by the fact that they rely on geometrical assumptions, on load dependency and have suboptimal reproducibility in different clinical scenarios [79]. STE is able to use strain imaging for measuring the RV myocardial deformation, mostly for detecting the subclinical phase of different diseases when other global parameters are still unchanged [79].

Mukherjee et al. demonstrated that even though conventional parameters routinely used to assess RV function (TAPSE, RVFAC) were similar between SSc patients and age- and sex-matched controls, RV free wall longitudinal strain (RVFWS) was significantly altered in the former, irrespective of the systemic disease subtype or sPAP [55]. Besides the absolute changes in the RVFWS value, it seems that there is a particular pattern as well, with higher reductions in the longitudinal strain value of the apical and middle segments and more efficient longitudinal function in the basal portions [55].

As such, RVFWS may be able to help clinicians identify occult contractile dysfunction and as a consequence, intensify the screening frequency of the SSc patients [79].

#### 6.3.5. Atrial Evaluation

As the majority of patients with SSc suffer from DD of the LV, not systolic dysfunction, it is imperative to be able to early detect the abnormality in the pattern of relaxation of the ventricle. LV DD is an independent predictor of all-cause mortality in the general population, even in the preclinical stage [80]. Unfortunately, the diagnosis of this clinical syndrome is not always straightforward. The algorithm proposed in 2016 for the echocardiographic evaluation of the LV diastolic function incorporates the maximum volume of the left atrium (LA), as it is easy to measure in most patients, but it also serves as a surrogate for the severity and chronicity of the elevated filling pressures of the left ventricle [81]. Adding to the value of volumetric measurement of the LA, assessment of the strain of the myocardial fibers in this cardiac chamber is able to detect earlier abnormal filling pressures of the LV as a marker of DD because changes in LA strain precede volumetric abnormalities [82].

From a general perspective, the LA has three basic functions, all of which have also been assessed in the systemic sclerosis population: reservoir (ε_r_), conduit (ε_CD_), and booster pump (ε_CT_) [83].

Myocardial fibrosis characteristic of SSc will lead to altered relaxation of the LV, which in turn will increase the filling pressures that, in the end, will generate fibrosis of the atrial myocardium. Changes in LA strain are correlated with the presence of fibrosis and LGE on CMR [82]. ε_R_ represents the LA function with the highest body of evidence from the literature regarding its usefulness in different clinical scenarios: in patients with atrial fibrillation, it correlates with age, LA size, and DD; it is a predictor of recovery of atrial contractility post-cardioversion and also a predictor of atrial fibrillation post transcatheter aortic valve replacement [84,85,86].

The first paper regarding changes in LA strain in patients with SSc was published in 2014 by Agoston et al., who compared 42 SSc patients with 42 healthy controls and observed that besides the fact that the first group had a higher E/e’ratio, ε_r_ and ε_CD_ were significantly reduced in SSc [87]. The authors conclude that altered LA mechanics, which can be observed in the absence of volumetric abnormalities, may represent an early sign of SScCmp [87].

In 2018, Porpaczy et al. observed that ε_r_ reduction in SSc mirrors the one exhibited by LV GLS, even in a SSc population without DD [88]. The other two parameters of LA function: ε_CD_ and ε_CT_ had reduced values in the SSc group with DD, but booster pump was reduced as well in patients with normal relaxation of the LV, with a mention that in grade 1 DD it increased as a consequence of the Frank-Starling mechanism [88]. One year later, the same group demonstrated that LA stiffness (which represents another parameter that further characterizes the LA function—it is defined as the change in pressure needed to increase the volume of the LA to a certain value) is superior in predicting high values of NTproBNP as a marker of LV DD than LA strain or volumetric measurement, with a value of 0.314 having the highest specificity [89].

Advanced assessment of LA function by multimodality imaging may provide further insight into the background of the sclerodermic process, but it also might be able to earlier detect subtle changes in myocardial function that should prompt the initiation of a more comprehensive cardiac screening program in certain SSc patients early.

**Figure 1:**
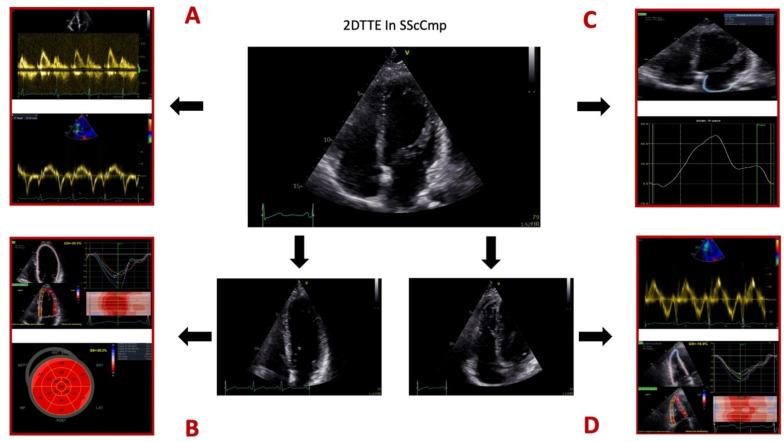
Different 2D transthoracic echocardiographic techniques used for evaluating the patients with systemic sclerosis and primary myocardial disease. Legend: Panel (**A**), mitral valve inflow profile by pulsed-wave (PW) Doppler, Tissue Doppler Imaging (TDI) for myocardial velocities. Panel (**B**), speckle-tracking echocardiography (STE) for assessing global longitudinal strain of the left ventricle (LV GLS). Panel (**C**), STE for assessing left atrial (LA) longitudinal strain. Panel (**D**), right ventricular (RV) evaluation comprised of TDI evaluation of RV free wall systolic velocity and RV free wall longitudinal strain. Images from the personal collection of the authors.

For a summary of different 2DTTE techniques employed for evaluating the SScCmp please see Figure 1.

### 6.4. CMR Assessment of Primary Myocardial Disease in SSc

Because of its high capacity of tissular characterization, CMR is a very accurate method for the non-invasive, non-irradiating evaluation of inflammation, perfusion and myocardial fibrosis [40]. Defining the multimodality imaging approach in the SScCmp, this diagnostic tool is able to identify cardiac disease (high T2 values, LV thinning, pericardial fluid, low LVEF and RVEF, LV diastolic dysfunction, LGE) in almost 75% of SSc patients [2,10,28,90].

CMR sequences bring complementary information about LV function, regional perfusion, angiogenesis, myocardial viability, and spatial disposition of the cardiomyocytes [58]. The superior sensitivity compared to echocardiography was confirmed by Hachulla et al. in a study of 52 SSc cases, in which 75% of them associated with at least one anomaly detectable by CMR, compared to only 28% by sole echocardiographic evaluation [56].

Large areas of fibrosis represent the hallmark of SSc and LGE is considered to be the “gold standard” for detection and characterization of focal myocardial fibrosis [28]. Unlike the one from ischemia or cardiomyopathies, SSc fibrosis is peculiar: its disposition is diffuse, in small intercellular quantities, and cannot be easily detectable with LGE in the initial phases of the disease [28,40]. Therefore, the lack of late enhancement on CMR does not exclude it, and because of that, a quantitative assessment is needed [28,40]. This implies measurement of fibrosis by T1 mapping and calculation of extracellular volume fraction (ECV), which will correlate with the degree of diffuse fibrosis [28]. Compared with controls, SSc patients associate higher mean T1 values, larger areas of myocardial damage detectable by native T1 mapping and larger ECV all in the absence of LV dysfunction [57].

Supplementary, in the setting of suspected myocardial inflammation, Markousis-Mavrogenis et al. demonstrated that one-quarter of symptomatic SSc patients from their study did not show any significant signs of an active inflammatory response on T2-based analysis [91]. In contrast, by T1-analysis, almost all patients with SSc and infective myocarditis displayed abnormalities, so the authors conclude that both T1 and T2 indices should be used in evaluating this specific category of patients [91].

Unlike subendocardial/transmural ischemic LGE, a patchy, mid-wall, linear pattern involving the basal and mid LV segments might be present in SSc [57]. Rodriguez-Reyna et al. observed myocardial fibrosis in 45% of 62 SSc cases, more prevalent in dcSSc and with disposition mostly in the basal parts of the LV [42]. It seems that fibrosis may be present before symptom onset and it affects the long-term prognosis of these patients [90].

Subclinical perfusion defects detectable by CMR are considered to be, alongside fibrosis, the dominant mechanism of cardiac injury in SSc [40]. The limitations generated by the disposition of the particular fibrosis pattern are also present in regard to the myocardial perfusion abnormalities: CMR absolute perfusion, traditionally used for their detection (most frequent in case of ischemia) is based on the relative differences between adjacent myocardial segments, so its utility is low in microvascular ischemia, specific to SSc [28,40]. Using models similar to the estimation of cardiac index by thermodilution, the absolute myocardial flow can be calculated by first-pass perfusion CMR, which identifies circumferential subendocardial ischemia [28].

Adenosine stress CMR is able to detect subendocardial perfusion defects in 79% of patients with SSc and cardiac disease [58].

In order to better describe the pathophysiologic background of myocardial damage in SSc, Mavrogeni et al. conducted a study on 46 recently diagnosed asymptomatic patients with dcSSc (45 female gender), mean age of 41 ± 5 years, with normal clinical examination, ECG and 2DTTE by using detection of myocardial edema and stress CMR [40]. Twenty healthy controls and 20 patients with angiographic established CAD were used for comparison [40]. Two cases had native T2 > 2 and the myocarditis protocol was positive [40]. The rest of 44 patients associated a T2 ratio < 2 and underwent stress-perfusion CMR for the detection of fibrosis [40]. Compared to healthy controls, all had a significant reduction in myocardial perfusion reserve index (MPRI), but similar with CAD cases, and the reduction was associated with the presence of digital ulcers [40]. Visual LGE was negative in all examinations, but quantitative analysis identified myocardial fibrosis in 40/44 SSc individuals (9.3 ± 8.7% of LV area), in larger quantities compared to healthy controls, but approximately the same with CAD cases, and the pattern was diffuse, respecting coronary vascular distribution [40]. There was no correlation between MPRI and duration/severity of the systemic disease [40]. A two-year follow-up was available in 11/44 cases and continuous asymptomatic reduction of MPRI was observed, alongside subendocardial LGE in 8/11 (10 ± 4% of LV mass), with normal LVEF [40]. The authors of the largest stress-perfusion fibrosis study for the evaluation of asymptomatic dcSSc compared to healthy and CAD controls, with follow-up data at 2 years in 25% of cases, confirm the previously published information, according to which, in this category of patients there is diffuse myocardial hypoperfusion, expressed by reduction of MPRI [40]. As such, low MPRI is an early indicator of myocardial damage in SSc, irrespective of the clinical status and the severity is similar to the one seen in CAD cases [40]. The follow-up evaluation from this study is suggestive for the way the fibrotic tissue progresses: at 2 years, cases presented diffuse subendocardial LGE, starting from an early stage, diffuse as well, but not detectable via late enhancement [40].

Regarding systolic and diastolic function, CMR-derived markers for its evaluation are: peak systolic circumferential strain, peak diastolic strain rate and increased volume of LA, all of them being much more prevalent in the SSc population compared to controls [57].

For the description of structural abnormalities involving the RV, CMR is considered to be the “gold standard”, allowing for accurate and reproducible measurements of ventricular dimensions, wall thickness and myocardial mass without relying on geometrical assumption [10]. Compared to other imaging methods, CMR identified altered function in a larger number of asymptomatic SSc cases [10].

### 6.5. Nuclear Imaging in Primary Myocardial Disease in SSc

The comprehensive evaluation of the SScCmp using a multimodality approach can offer a complete description of the subtle changes undergone by the heart and is important at least in the research setting. Nuclear imaging-SPECT, PET-CT—is recommended for assessing myocardial perfusion dysfunction [58].

Thallium-201 perfusion scintigraphy, together with SPECT, detects reversible myocardial perfusion defects induced by cold stimulus or physical stress, in almost all SSc cases [10]. These are more frequently seen in severe, rapid progressive cutaneous disease, with immunologic profile dominated by the presence of anti-Scl70 antibodies [10]. Detection of perfusion defects by means of nuclear imaging can identify a certain subgroup of SSc cases that associates a more severe systemic disease phenotype [10].

Follansbee et al. described a group of dcSSc cases without symptomatic heart disease that on post physical stress thallium scans presented a high rate of myocardial perfusion defects, in the absence of significant CAD [92].

In SSc, one of the responsible mechanisms for myocardial injury and subsequent fibrosis is represented by the transitory coronary spasm induced by different stimuli. Moreover, even though CMR distinguishable fibrosis has large distribution, it seems that vascular spasm detectable by nuclear imaging leads to ischemia of large myocardial areas, since it is necessary for more than 5 g of hypoxic tissue/ventricular for thallium scintigraphy to detect it [17].

Using thallium 210-SPECT, Kahan et al. reported that almost 20% of analyzed ventricular segments presented an uptake defect, detectable at rest and partly reversed after administration of dipyridamole, nicardipine or nifedipine [93,94,95]. Assessing 48 SSc cases, Steen et al. observed that myocardial perfusion defects represent one of the most important predictors for future myocardial damage and mortality, irrespective of the cutaneous subtype [10,18,29].

**Figure 2:**
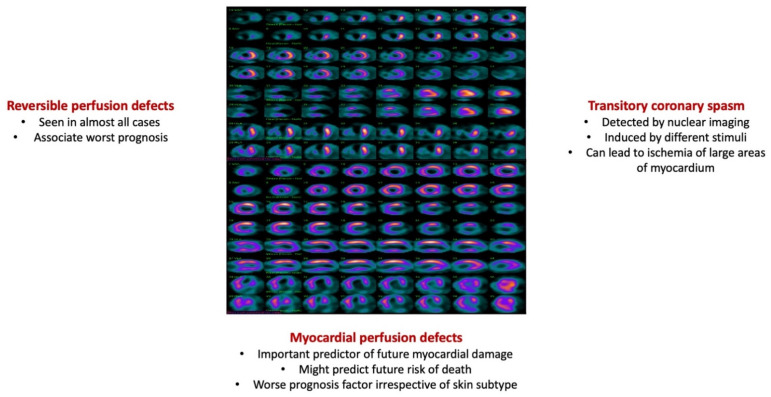
Most important findings by nuclear imaging in patients with systemic sclerosis and primary myocardial disease. Images from the personal collection of the authors.

Figure 2 summarizes the most important nuclear imaging findings in systemic sclerosis-associated primary myocardial disease.

## 7. Outcomes of Primary Myocardial Involvement in SSc

The heart is one of the most important internal organs affected in SSc and is responsible for a large number of deaths in these patients (15%), while over the years, the rate of pulmonary and renal complications has declined gradually [58,96]. From the moment symptoms start developing, cardiac affliction becomes a major predictor of mortality (survival rates of 40 and 24% at 2 and 5 years, respectively) [11,15]. However, as pulmonary fibrosis can, in turn, lead to pulmonary hypertension and cor pulmonale, it is sometimes difficult to distinguish the primary and secondary cardiac involvement, and multimodality cardiac imaging brings an essential contribution.

**Figure 3:**
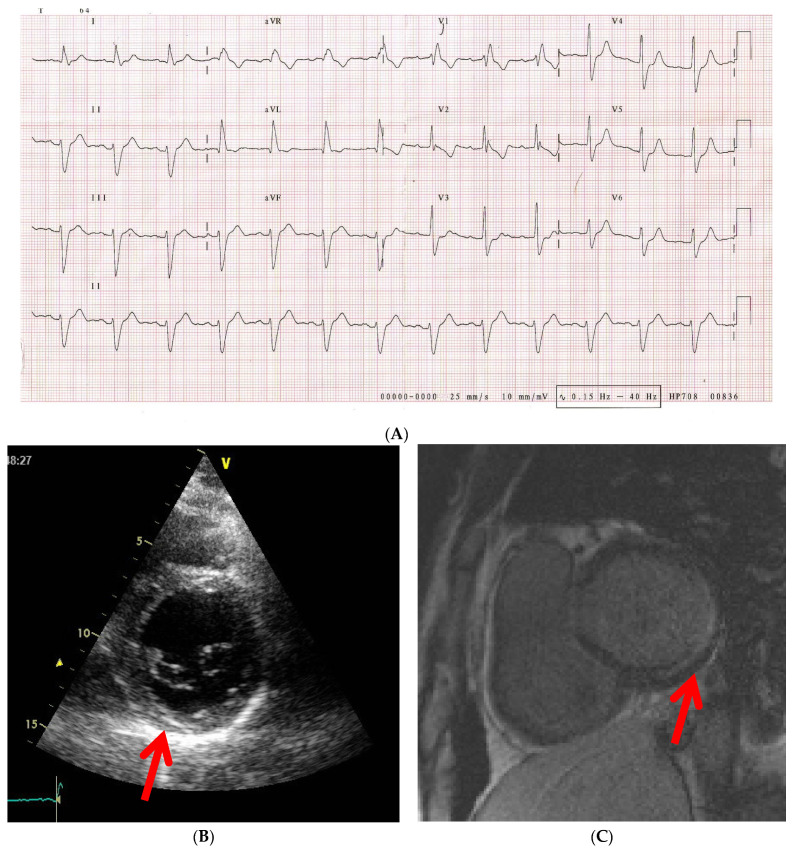
42 years old male patient diagnosed with dcSSc, normal epicardic coronary arteries. (**A**) ECG tracing with trifascicular block (RBB, AFB, grade 1 AV block), (**B**) 2DTTE PSAX image at the base of the LV showing hyperechogenic area in the infero-basal segment of the LV (red arrow); (**C**) CMR image showing LGE in the infero-basal segment suggestive of subendocardial and mid-wall scar at this level (red arrow). dcSSc, diffuse cutaneous systemic sclerosis; RBB, right bundle branch block; AFB, anterior fascicular block; AV, atrio-ventricular; 2DTTE, two-dimensional transthoracic echocardiography; PSAX, parasternal short-axis view; LV, left ventricle; CMR, cardiac magnetic resonance; LGE, late gadolinium enhancement.

Overt cardiac disease is seen in 15–35% of cases, but in morpho-pathological studies it is identified in 21–100% of situations, and most patients suffer from subclinical disease [44,58] (Figure 3). Still, there are reasons to believe that the true prevalence is much higher, given the fact that signs and symptoms are occult, heart pathology tends to coexist with pulmonary, musculoskeletal and esophageal comorbidities, which can lead to false identification of symptoms and diagnostic tests have relative inaccuracy [10,11].

Although primary myocardial dysfunction is not seen as frequently as pericardial disease or arrhythmia, it is a major predictor of mortality, making early detection crucial [10,23,97]. Heart disease in SSc is a clue for an aggressive phenotype of the systemic pathology, it increases mortality risk five times, and 5-year mortality rates regarding primary myocardial involvement reach up to 70%, with global cardiac affliction representing the cause of 25–30% of total deaths in the SSc population [2,10,14,96]. SSc patients appear to have twice the need for ICD/pacemaker implantation compared to cases without SSc [30,98].

After a 10-year follow-up of 953 dcSSc cases, Medsger and Steen reported heart-related mortality of 20%, and in an Italian cohort of 1012 patients, heart disease solely accounted for up to 36% of deaths [18,32,99]. Moreover, a retrospective analysis conducted by Vlachoyiannopoulos et al. on 254 SSc individuals provided data according to which annual mortality rate related to cardiac involvement was 2% and the incidence varied only slightly with the cutaneous subtype: 7% lcSSc and 21% dcSSc [100]. Another review (between 1959–1988) focused on 1095 patients with SSc and reported a mortality rate because of cardiac disease of 4.5% from a total of 33% general mortality [44,62].

Overall, cardiac comorbidity is responsible for 20% of total deaths in the SSc population and data from the Spanish Registry of Systemic Sclerosis demonstrates that the relative risk of mortality is superior to the one related to pulmonary or renal causes (2.8 vs 1.9 and 1.6) [30,101]. After analyzing 11193 cases from EUSTAR, Elhai et al. incriminated cardiac cause as the main reason for mortality in 27% of cases [102]. Keeping in mind that conventional CVRF can also contribute to cardiac mortality, screening for their presence and optimizing their control remains of utmost importance, together with active SScCmp screening.

## 8. Treatment of SScCmp

Presently, there is no specific treatment algorithm for SScCmp [103,104]. In general, cardiac dysfunction in this population is managed according to the existing guidelines [57,104].

Patients with SSc and HFrEF should benefit from initiation of guideline directed medical therapy consisting from beta-blockers (βb), angiotensin-converting enzyme inhibitors (ACEi)/angiotensin receptor blockers (ARB) and mineralocorticoid receptor antagonists (MRA) [57]. Suffering from a chronic autoimmune disorder, it has been postulated that SSc cases could receive earlier therapeutic intervention than the general population [97].

CCB lowers the risk of LV systolic dysfunction and also leads to improvement in cardiac function and perfusion detectable by SPECT, 2DTTE, and CMR [52,95,105,106]. ACEi/ARB and CCB decrease the risk of ventricular arrhythmias, and low dose aspirin is associated with a reduction of conduction abnormalities and pacemaker implantation [13].

If suspected, CAD should be detected and treated as in the general population [57].

Medium dose oral corticotherapy improves LV function, but also, together with immunosuppressive agents, represents an option in SSc cases with myocardial fibrosis that exhibit progressive worsening of cardiac function, despite adequate therapy [107,108]. Pulse corticosteroids decrease myocardial edema detectable by CMR [109].

Because myocarditis can complicate the clinical evolution of SScCmp, the clinician must be aware of the fact that immunosuppresive agents (cyclophosphamide, mycophenolate mofetil, azathioprine) used in these specific cases have led to clinical, cardiac biomarkers and CMR improvement [15,108,110].

In cases of advanced HF, cardiac transplantation, cardiac resynchronization therapy, and mechanical support devices should be used according to standard guidelines [57].

## 9. Conclusions

Generated by a polymorphic systemic disease with very complex pathogenesis and intricated chronic mechanisms, SScCmp is an underrecognized and underdiagnosed clinical entity. However, the recent advancement of imaging techniques continues to pave the road started decades ago by nuclear radiology, in showing that the intricated interplay between microangiopathy and fibrosis continues to express itself at a myocardial level.

The rate of adverse clinical outcomes, of systolic/diastolic dysfunction, conduction/arrhythmic disturbances, or even of SCD can be improved by a multimodality cardiovascular evaluation of the SSc patient for timely diagnosis of myocardial involvement.

## Figures and Tables

**Table 1:** Summary of cardiac investigations used for diagnosis and follow-up in the sclerodermic cardiomyopathy.

Investigation	Main Findings
**Cardiac biomarkers**	**NTproBNP**—first-line diagnostic tool; follow-up; does not vary across skin subtypes [23]. Cut-off value of 130 pg/mL-sensitivity 74%, specificity 70%, NPV 85% for detecting cardiac impairment in SSc [23] **cTn**—positive correlation with cardiac disease in SSc, but with low specificity and sensitivity [46]
**ECG**	25–75% SSc patients have abnormal tracings [39]. Independent predictor of mortality [39]. Anteroseptal myocardial infarction pattern—10% of cases [30]. High frequency of arrhythmias—especially ventricular events [47]. High frequency of PVB—correlates with mortality and SCD risk [48]; >1190 PVB/24 h—100% sensitivity and 83% specificity in predicting SCD or later necessity of ICD implantation [49]. Presence/severity of arrhythmias does not correlate with skin subtype/symptoms [39]. Arrhythmogeneity index—correlates with mRSS [50].
**.2DTTE**	SSc patients need annual 2DTTE for assessing LV systolic and diastolic function and sPAP [51]. **LV systolic function:** 5.4% prevalence of LV systolic dysfunction [52]; reduced strain rate values in the IVS and antero-lateral wall [12]; myocardial electromechanical proprieties—diffuse deterioration of LV segments [3]. **LV diastolic function:** 4–5 times more frequent than systolic dysfunction (17–30% of cases) [22,53]; Risk factors for low e’velocities: duration of the disease, age, CAD, HTN [53]; e’-baseline value predicts mortality risk and every decrease in standard deviation elevates mortality risk 3.2 times [53]. **VAC:** Higher in dcSSc compared to lcSSc [51]; VAC might predict MACE in SSc population [51]. **RV:** Frequently involved in systemic diseases, either by direct injury, or because of associated PH [54]. Early dysfunction in SSc—associated with the degree of skin and pulmonary involvement and with anti-Scl70+ [12,54]. Early diastolic dysfunction [54]; prolongation of pre-ejection times [54]; RVFWS—significantly lower in SSc cases compared to healthy controls (irrespective of skin subtype or sPAP) [55]; apical and middle segments of RVFW—more damaged compared to the basal segment [55].
**CMR**	Detects cardiac disease in up to 75% of SSc cases, superior sensitivity than echocardiography for detecting cardiac abnormalities in SSc [56]. Large areas of fibrosis with specific disposition: diffuse, in small intercellular quantities [28]; higher T1 values and larger ECV compared to controls [57]; patchy, mid-wall, linear pattern in the basal and mid-LV segments [57]; fibrosis more prevalent in dcSSc [42]. Adenosine stress CMR–79% SSc cases with cardiac disease have subendocardial perfusion anomalies [58].
**Nuclear Techniques**	Thallium-201 perfusion scintigraphy +SPECT—detect reversible induced myocardial perfusion defects in almost all patients [10]. Perfusion defects more prevalent in severe cutaneous disease and anti-Scl70+ [10]. Myocardial perfusion defects—one of the most important predictors for future myocardial damage and mortality, irrespective of skin subtype [10,18,29].

Legend: NTproBNP, N-terminal pro B-type natriuretic peptide; NPV, negative predictive value; SSc, systemic sclerosis; cTn, cardiac troponin; ECG, electrocardiography; PH, pulmonary hypertension; PVB, premature ventricular beats; SCD, sudden cardiac death; ICD, implantable cardioverter-defibrillator; mRSS, modified Rodnan skin score; 2DTTE, two-dimensional transthoracic echocardiography; LV, left ventricle; sPAP, systolic pulmonary artery pressure; IVS, interventricular septum; CAD, coronary artery disease; HTN, arterial hypertension; VAC, ventricular-arterial coupling; dcSSc, diffuse cutaneous systemic sclerosis; lcSSc, limited cutaneous systemic sclerosis; MACE, major adverse cardiac events; RV, right ventricle; anti-Scl70, anti–topoisomerase 1 antibody; RVFWS, RV free wall strain; CMR, cardiac magnetic resonance; ECV, extracellular volume; SPECT, single photon emission computed tomography.

## Data Availability

Not applicable.
